# Case Report: Endobronchial antibiotic instillation for refractory lung abscess due to *Streptococcus pyogenes*

**DOI:** 10.3389/fmed.2026.1847731

**Published:** 2026-06-10

**Authors:** Jennifer A. Doran, Kelli A. Keene, Michael A. Miller, Travis L. Pollema, Mazen F. Odish, Ajay R. Bharti, Chelsea E. Roche, Elizabeth K. Weihe, Keriann M. Van Nostrand, Omar A. Mesarwi

**Affiliations:** 1 Department of Medicine, Division of Pulmonary, Critical Care, Sleep Medicine and Physiology, University of California, San Diego, San Diego, CA, United States; 2 Skaggs School of Pharmacy and Pharmaceutical Sciences, University of California, San Diego, San Diego, CA, United States; 3 Division of Cardiovascular and Thoracic Surgery, Department of Surgery, University of California, San Diego, San Diego, CA, United States; 4 Division of Infectious Diseases and Global Public Health, Department of Medicine, University of California, San Diego, San Diego, CA, United States; 5 Department of Radiology, University of California, San Diego, San Diego, CA, United States; 6 Section of Sleep Medicine, Jennifer Moreno Department of Veteran Affairs Medical Center, San Diego, CA, United States

**Keywords:** ARDS (acute respiratory disease syndrome), bronchoscopic administration, endobronchial antibiotics, pneumonia, pulmonary abscess, refractory abscess, *Streptococcus pyogenes*

## Abstract

Lung abscesses can be difficult to treat despite long antibiotic courses, and treatment failure is frequently observed. Percutaneous drainage or surgical resection may be considered in select patients, and intracavitary antimicrobial instillation has been described for treatment failure in highly selected cases. We present a case of an immunosuppressed patient with acute respiratory distress syndrome who required mechanical ventilation and venovenous extracorporeal membrane oxygenation due to *Streptococcus pyogenes* pneumonia with a large intrapulmonary abscess. The abscess was refractory to appropriate systemic antibiotic therapy, and direct intracavitary instillation was not feasible, so serial endobronchial antimicrobial lavage near the abscess site was used as salvage therapy to treat the abscess. Radiographic and clinical improvement were noted soon thereafter, without any observed adverse effects. Proximate endobronchial antimicrobial therapy was well tolerated and may be useful in additional cases of refractory pulmonary abscess. In this report, we describe the rationale and considerations underlying the choice of antimicrobial therapy.

## Introduction

Lung abscesses can develop from pneumonia, causing cavitation and necrosis. Treatment usually requires prolonged antibiotic courses but fails in 10–20% of cases. Failure can be attributed to poor antibiotic penetration or underlying lung disease preventing drainage and source control, necessitating further intervention such as surgical resection or percutaneous drainage (1). Many patients are not suitable candidates for invasive interventions due to critical illness; however, mortality rates remain high at 15 to 20% (1). Intracavitary antimicrobial instillation has been described for the treatment of aspergillomas (2, 3), but there are limited data regarding the use of endobronchial antibiotics for bacterial lung abscesses. We present a case of severe *Streptococcus pyogenes* (*S. pyogenes*) pneumonia with a large pulmonary abscess, in which clindamycin was instilled near the abscess as an adjunct to systemic antibiotics. We provide the rationale and key practical considerations for this technique.

## Case report

**Figure 1:**
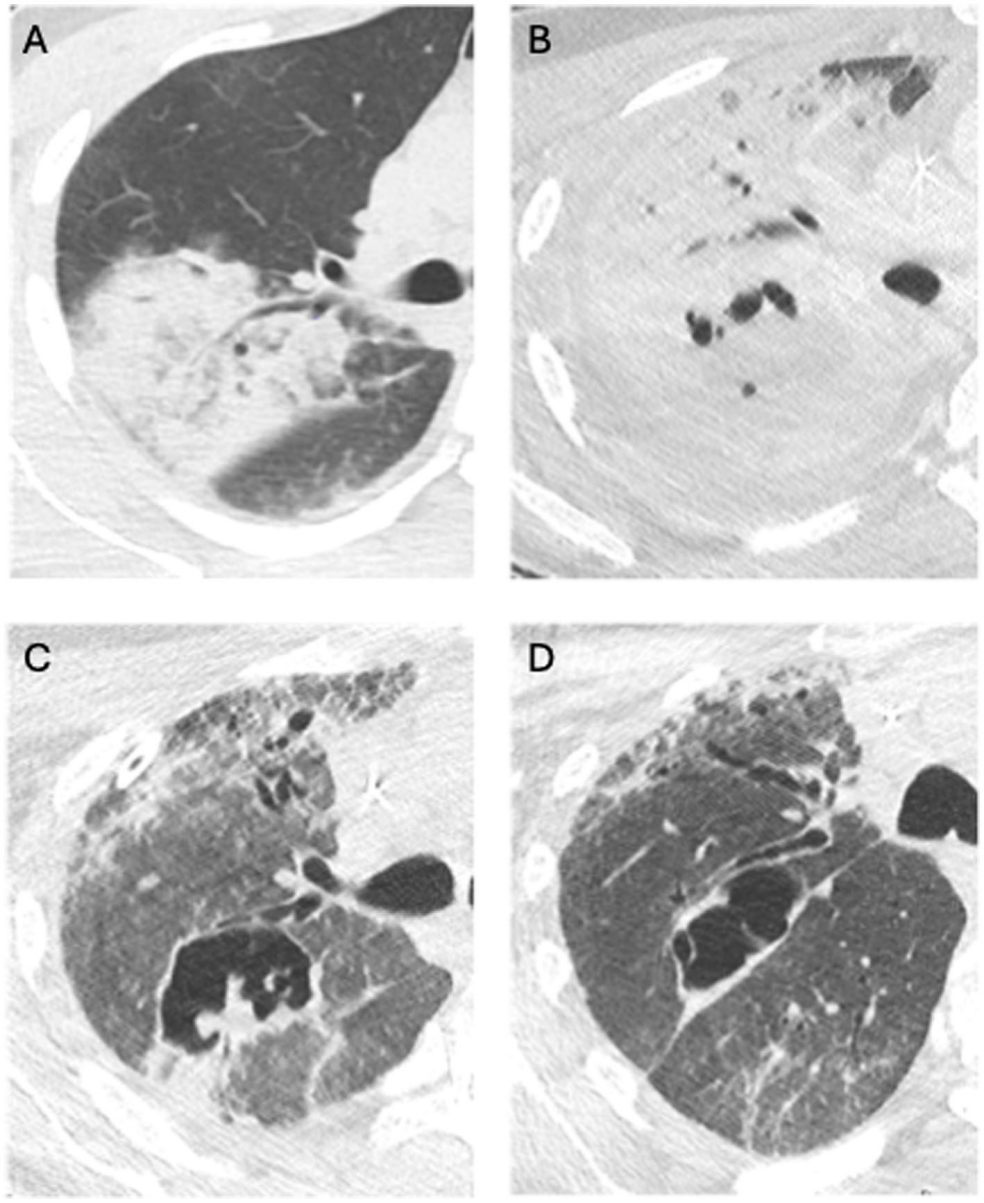
Progression of pulmonary abscess over the patient’s hospital course. **(A)** Hospital Day 1, initial presentation showing right upper lobe abscess with surrounding pneumonia. **(B)** Hospital Day 13, with multi-loculated, fluid-filled abscess and severe surrounding pneumonia and ARDS. **(C)** Hospital Day 24, after intrabronchial clindamycin. The abscess is more organized, and less fluid-filled, with early fibrotic changes. **(D)** Hospital Day 31, demonstrating cavitation and very little fluid component in the abscess space. ARDS, acute respiratory distress syndrome.

We present the case of a 35-year-old man with rheumatoid arthritis who was receiving methotrexate and hydroxychloroquine and presented with fever, cough, and diarrhea. He described a recent trip to India to visit family. An upper respiratory viral swab was positive for influenza A, and *S. pyogenes* quickly grew in blood cultures. His initial presentation was consistent with septic shock, and he was initially treated with oseltamivir, ceftriaxone, azithromycin, intravenous vasopressors, and corticosteroids for refractory shock. His respiratory status worsened, and on hospital day 5, he developed a spontaneous secondary right tension pneumothorax that required immediate tube thoracostomy and mechanical ventilation for acute respiratory distress syndrome (ARDS). His hypoxemia progressively worsened, requiring venovenous extracorporeal membrane oxygenation (ECMO) and neuromuscular blockade the following day. Antimicrobials were broadened to meropenem and micafungin due to the severity of his illness and concern for insufficient antimicrobial coverage. On hospital day 13, chest computed tomography (CT) showed worsening pneumonia with progression of a right upper lobe (RUL) abscess (Figures 1A,B). Bronchoalveolar lavage cultures and Karius testing identified only *S. pyogenes*, which was pan-susceptible on antimicrobial susceptibility testing. With no improvement after 2 weeks of appropriate intravenous antibiotics, there was concern for poor antibiotic penetration into the abscess. Multidisciplinary discussions involving specialists from critical care, interventional pulmonology, radiology, infectious diseases, and pharmacy led us to consider endobronchial instillation of clindamycin near the abscess site, in addition to ongoing intravenous antibiotics, as invasive interventions were not feasible given his ongoing critical status.

On hospital days 15–18, the patient had clindamycin instilled into the RUL segmental airways (600 mg diluted into 180 mL of normal saline) as follows: An Arndt 7-French endobronchial blocker was placed into the target airway near the abscess, and the balloon was carefully inflated under direct visualization. An adequate seal was confirmed with a test instillation of 5 mL of sterile saline. Then, clindamycin was delivered in three 60-mL aliquots, while monitoring for evidence of leakage. The blocker remained inflated with direct visualization for 15 min, allowing the antibiotic time to dwell and be absorbed into the lung parenchyma. During this time, intravenous antibiotics were narrowed to penicillin G since no other organisms grew in cultures. The patient’s lung compliance improved concurrently with these changes. On hospital day 23, neuromuscular blockade was discontinued, and the following day, a repeat chest CT revealed improvement of the abscess and lung aeration (Figure 1C). Leukocyte count normalized soon thereafter. On hospital day 39, he was decannulated from ECMO, and a week later, he was discharged from the hospital on oral amoxicillin for an additional 2 weeks. Prior to discharge, CT showed continued improvement in lung consolidation and the abscess (Figure 1D).

## Discussion

We present a case of severe ARDS due to *S. pyogenes* complicated by a pulmonary abscess refractory to appropriate intravenous antibiotics. As the patient had severely injured lung parenchyma due to pneumonia, there was concern that IV antibiotics were unable to adequately penetrate the lung abscess and support recovery. Percutaneous drainage was not deemed a viable option because the abscess was multi-loculated, poorly defined, and more centrally located (1, 4). These factors, especially in the setting of surrounding severe lung injury, made the risk of associated complications, including bronchopleural fistula, pneumothorax, and failed drainage, too high (4). Surgical resection was also considered too risky due to the severity of the patient’s illness. After 2 weeks without improvement despite appropriate intravenous antimicrobial therapy, endobronchial instillation of antibiotics into the most accessible bronchi near the abscess was attempted. We believe that this atypical treatment course contributed significantly to the patient’s subsequent improvement.

We considered several issues when selecting a protocol for proximate endobronchial antibiotic delivery, involving consultation with a multidisciplinary team of experts. First, we sought an antibiotic that was known to be effective against *S. pyogenes*. Second, anticipating limited time to dwell on the antibiotic each day, we wanted a drug with at least partial concentration-dependent activity rather than solely time-dependent activity (5). Third, we wanted to ensure safety with direct endobronchial administration, so we tested the pH of the antibiotic before administration and of the aspirated fluid after dwell (7.2 and 7.4, respectively). Finally, we needed an antibiotic that could be delivered in a volume appropriate for segmental lavage. Clindamycin, a lincosamide antibiotic that functions by binding to the bacterial 50S ribosomal subunit, thereby inhibiting protein synthesis, fulfilled these criteria (6). Clindamycin is primarily bacteriostatic with bactericidal properties at higher concentrations and has time-dependent but concentration-enhanced activity with a significant post-antibiotic effect, providing rationale for our use of this medication even with limited dwell times (7, 8). Moreover, the bacteriostatic effect of clindamycin can act on exotoxins even at suboptimal concentrations (9). This mechanism is thought to be a particularly important defense against organisms such as *S. pyogenes*. Finally, clindamycin is known to penetrate abscess walls readily, which was particularly helpful in this case.

Direct instillation of endobronchial antifungals is well documented for pulmonary aspergilloma, with anywhere from 2 to 9 sessions associated with improved outcomes in several case series (10) and in a single randomized controlled trial (2). Regarding pulmonary abscesses, percutaneous drainage is sometimes used in select patients when surgical resection is not feasible (11). Endobronchial antibiotic administration in cases of pulmonary abscesses has also been described in other studies, but only when the abscess cavity is bronchoscopically accessible and can be drained and subsequently washed with antibiotics (12–14). In our patient, it was radiographically difficult to ascertain an ideal airway communication to the abscess since it was multiloculated, so clindamycin was instilled near, but not directly into, the abscess itself. Our decision to treat for a total of four sessions was based on daily discussions with our multidisciplinary team about the risks and benefits of our intervention. By day 4 of instillation, we felt that we were already seeing signs of improvement and that the risk of further procedures might not be warranted. Nonetheless, the duration of treatment requires further study.

Although we cannot draw causative inferences about the relationship between the patient’s clinical improvement and our intervention, it appeared to be safe, well tolerated, and temporally associated with a clinical inflection point in recovery. In conclusion, proximate endobronchial antimicrobial instillation may be useful in the treatment of refractory pulmonary abscesses, with careful consideration of the rationale for case presentation, patient selection, and antimicrobial choice.

## Data Availability

The original contributions presented in the study are included in the article/supplementary material, further inquiries can be directed to the corresponding author.

## References

[1] Wali SO. An update on the drainage of pyogenic lung abscesses. Ann Thorac Med. (2012) 7:3–7. doi: 10.4103/1817-1737.91552, 22347342 PMC3277038

[2] Hadda V Doddamani S Mittal S Tiwari P Madan K Mohan A . Efficacy of Intrabronchial Voriconazole instillation for inoperable pulmonary Aspergilloma: a pilot randomized controlled trial. Respiration. (2022) 101:833–40. doi: 10.1159/000525376, 35810744

[3] Kravitz JN Berry MW Schabel SI Judson MA. A modern series of percutaneous intracavitary instillation of amphotericin B for the treatment of severe hemoptysis from pulmonary aspergilloma. Chest. (2013) 143:1414–21. doi: 10.1378/chest.12-1784, 23117277

[4] Jorge PC Thomas HM Arshad S. Large lung abscesses managed with percutaneous drainage. Brown J Hosp Med. (2022) 1:37844. doi: 10.56305/001c.37844, 40046582 PMC11878840

[5] Levison ME Levison JH. Pharmacokinetics and pharmacodynamics of antibacterial agents. Infect Dis Clin N Am. (2009) 23:791–815, vii. doi: 10.1016/j.idc.2009.06.008, 19909885 PMC3675903

[6] Murphy PB Bistas KG Patel P Le JK. Clindamycin. In: StatPearls. Treasure Island (FL): StatPearls Publishing. Available online at: https://www.ncbi.nlm.nih.gov/books/NBK519574/

[7] Levison ME. Pharmacodynamics of antimicrobial drugs. Infect Dis Clin N Am. (2004) 18:451–65. doi: 10.1016/j.idc.2004.04.012, 15308272

[8] Pichereau S Moran JJ Hayney MS Shukla SK Sakoulas G Rose WE. Concentration-dependent effects of antimicrobials on *Staphylococcus aureus* toxin-mediated cytokine production from peripheral blood mononuclear cells. J Antimicrob Chemother. (2012) 67:123–9. doi: 10.1093/jac/dkr417, 21980070

[9] Sawai J Hasegawa T Kamimura T Okamoto A Ohmori D Nosaka N . Growth phase-dependent effect of clindamycin on production of exoproteins by *Streptococcus pyogenes*. Antimicrob Agents Chemother. (2007) 51:461–7. doi: 10.1128/AAC.00539-06, 17101685 PMC1797754

[10] Sharma S Kumar R Ish P Mahendran AJ Gupta NK Gupta N . Clinical utility of intrabronchial antifungal instillation in a complicated case of chronic pulmonary aspergillosis: case report and systematic review of literature. Infez Med. (2023) 31:575–82. doi: 10.53854/liim-3104-17, 38075417 PMC10705858

[11] Lee JH Hong H Tamburrini M Park CM. Percutaneous transthoracic catheter drainage for lung abscess: a systematic review and meta-analysis. Eur Radiol. (2022) 32:1184–94. doi: 10.1007/s00330-021-08149-5, 34327579

[12] Hadid W Stella GM Maskey AP Bechara RI Islam S. Lung abscess: the non-conservative management: a narrative review. J Thorac Dis. (2024) 16:3431–40. doi: 10.21037/jtd-23-1561, 38883669 PMC11170420

[13] Ainge-Allen HW Lilburn PA Moses D Chen C Thomas PS. Antibiotic instillation for a chronic lung abscess. Respir Med Case Rep. (2020) 29:100991. doi: 10.1016/j.rmcr.2019.100991, 31908918 PMC6940720

[14] Cascone R Sica A Sagnelli C Carlucci A Calogero A Santini M . Endoscopic treatment and pulmonary rehabilitation for Management of Lung Abscess in elderly lymphoma patients. Int J Environ Res Public Health. (2020) 17. doi: 10.3390/ijerph17030997, 32033391 PMC7038113

